# Sites of persistence of *Fusobacterium necrophorum* and *Dichelobacter nodosus*: a paradigm shift in understanding the epidemiology of footrot in sheep

**DOI:** 10.1038/s41598-019-50822-9

**Published:** 2019-10-08

**Authors:** Rachel Clifton, Katharina Giebel, Nicola L. B. H. Liu, Kevin J. Purdy, Laura E. Green

**Affiliations:** 1 School of Life Sciences, University of Warwick, Coventry, CV4 7AL UK; 2 Present Address: College of Life and Environmental Sciences, University of Birmingham, Edgbaston, B15 2TT UK; 3 Present Address: QMMS Limited, Wells, UK

**Keywords:** Microbial ecology, DNA replication, Infectious-disease epidemiology

## Abstract

Sites of persistence of bacterial pathogens contribute to disease dynamics of bacterial diseases. Footrot is a globally important bacterial disease that reduces health and productivity of sheep. It is caused by *Dichelobacter nodosus*, a pathogen apparently highly specialised for feet, while *Fusobacterium necrophorum*, a secondary pathogen in footrot is reportedly ubiquitous on pasture. Two prospective longitudinal studies were conducted to investigate the persistence of *D. nodosus* and *F. necrophorum* in sheep feet, mouths and faeces, and in soil. Molecular tools were used to detect species, strains and communities. In contrast to the existing paradigm, *F. necrophorum* persisted on footrot diseased feet, and in mouths and faeces; different strains were detected in feet and mouths. *D. nodosus* persisted in soil and on diseased, but not healthy, feet; similar strains were detected on both healthy and diseased feet of diseased sheep. We conclude that *D. nodosus* and *F. necrophorum* depend on sheep for persistence but use different strategies to persist and spread between sheep within and between flocks. Elimination of *F. necrophorum* would be challenging due to faecal shedding. In contrast *D. nodosus* could be eliminated if all footrot-affected sheep were removed and fade out of *D. nodosus* occurred in the environment before re-infection of a foot.

## Introduction

Footrot is one of the top 5 globally important diseases of sheep^1–3^, causing lameness, poor health and welfare^4^ and decreased productivity^5–7^. It costs the UK sheep industry between £20 and £80 million per annum^1^, hence affecting the sustainability of sheep farming. There are two disease presentations of footrot: interdigital dermatitis (ID) characterised by an inflammation of the epidermal interdigital skin which may progress to severe footrot (SFR) characterised by separation of the hoof horn from underlying tissues^8^.

Footrot is present in all countries with domesticated sheep, however, disease expression is highly influenced by soil moisture and so footrot presents differently in different climates. Transmission of footrot occurs between sheep in damp conditions^9,10^ but not in dry, hot or very cold conditions^3^. Consequently, in regions with extreme climates, for example, Western Australia, South India and Switzerland there are prolonged periods of non-transmission of footrot^11–13^ and footrot can ‘disappear’ from a flock. In temperate climates, such as in the UK and Ireland, disease expression is continuous with epidemics typically in the wetter weather of spring and autumn^10,14,15^.

There are many bacterial species on the feet of sheep, both healthy and footrot affected^8,16^, however, footrot is caused by *Dichelobacter nodosus*^8,17,18^, a Gram-negative anaerobe which is highly adapted to the ovine foot^19^ and is key in the initiation of ID and progression to SFR^18,20^. *Fusobacterium necrophorum* is often present as a secondary pathogen that increases disease severity^18,21^. Feet with footrot can remain diseased for several months^8,22,23^, although it is not known if this is a persistent infection or repeated re-infections. It has been hypothesised by observation of disease development, that *D. nodosus* persists on pasture for only a few hours to a few days^24,25^. In contrast to *D. nodosus*, *F. necrophorum* is an opportunistic pathogen that causes necrotic lesions at a range of anatomical sites and in many host species^26–28^. It is widely reported in veterinary textbooks, reviews, and primary research papers^15,27–31^ that *F. necrophorum* is shed in faeces of ruminants and consequently persistently present on pasture, from where it invades the interdigital skin. To date much of the above is speculation or from challenge studies between diseased and non-diseased sheep, *D. nodosus* and *F. necrophorum* have been detected on healthy feet^18,32,33^ and in mouths of sheep, in cross sectional studies^34,35^, but it is not known whether these sites are reservoirs of *D. nodosus*. To date, there have been no longitudinal molecular epidemiology studies to investigate the sites of persistence of *D. nodosus* and *F. necrophorum* on sheep and in the farm environment.

The aim of the current study was to use two longitudinal studies of two sheep flocks during different climates in the UK to identify sites of persistence of *D. nodosus* and *F. necrophorum* in sheep and their environment. Elucidating where these organisms persist would improve our understanding of the epidemiology of footrot and so inform on the probability of elimination or control of these pathogens.

## Materials and Methods

### Ethical approval

Ethical approval for the studies was obtained from the University or Warwick’s local ethical committee; the Animal Welfare & Ethical Review Body (AWERB.33/13-14). Faecal sampling was carried out under Home Office Licence (PPL 70/8392). All experiments were performed in accordance with relevant guidelines and regulations.

### Study populations, study samples and data collected for Studies 1 and 2

**Figure 1:**
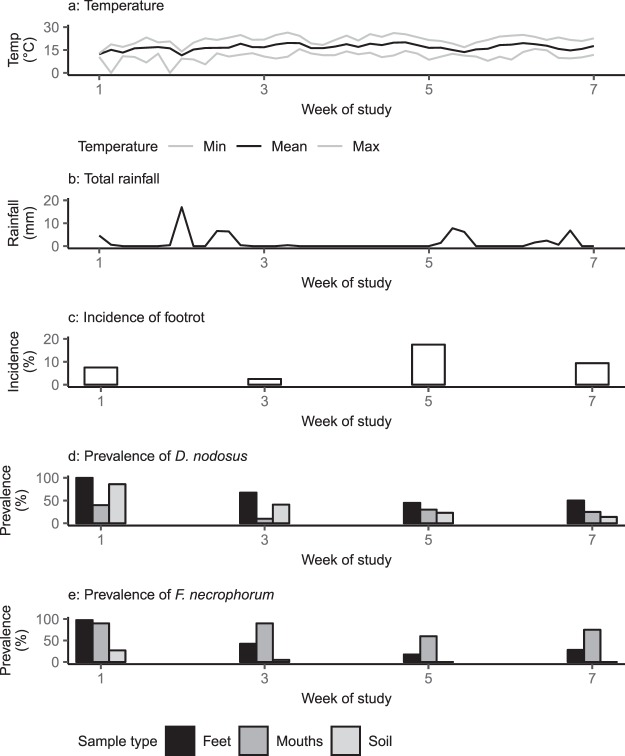
(**a**) Temperature; (**b**) total rainfall; (**c**) incidence of footrot; (**d**) prevalence of *Dichelobacter nodosus*; (**e**) *Fusobacterium necrophorum* by week for Study 1. (**a**) Temperature (°C). Black = mean, grey = minimum and maximum temperatures; (**b**) total rainfall (mm); (**c**) incidence of footrot as a percentage of feet examined; (**d**) prevalence (%) of *Dichelobacter nodosus* as a percentage of total number of samples for each sample type by week for study 1*;* (**e**) prevalence (%) of *Fusobacterium necrophorum* as a percentage of total number of samples for each sample type by week for Study 1.

Study 1 was conducted on a lowland (altitude approximately 88 meters above sea level with agricultural land classification 2 and 3^36^) commercial sheep farm in Warwickshire, England during a period of warm, wet weather with footrot transmission (Fig. 1). The flock was comprised of approximately 150 2–5-year-old North-Country Mule x Texel ewes and their 3–6-month-old lambs and was selected by farm location, farmer compliance and footrot in the flock. A group of 40 sheep were gathered at the first visit and four lame sheep (two ewes and two lambs) and six non-lame sheep (three ewes and three lambs), the 10 study sheep, were selected using convenience sampling^37^. Lambs were unrelated to ewes. The whole group was kept on one pasture for the duration of the study. The 10 study sheep were sampled at 2-week intervals on four occasions between May and July 2014. All samples were collected by Rachel Clifton and Katharina Giebel. Each sampling was comprised of scoring the footrot phenotype of feet^38^ and taking swab samples from the interdigital skin of all four feet and the gingival crevice; swab samples were taken by making 5 swipes down the interdigital skin or across the gingiva. The pasture was divided into two high traffic areas, an open gateway and a shaded area under a tree, where sheep regularly stood or laid down in close proximity and the remaining pasture was a low traffic area where sheep were rarely present. The two high traffic areas were sampled at their centre, and at 1 m and 2 m radii. The low traffic area of the field was sampled randomly at five locations at each visit; a 20 m × 20 m grid with 25 × 5 m intersections of the grid were used to select sampling points^39^. At each sample point a soil corer (diameter 3.5 cm) was used to collect two soil samples at 0–1 cm and 4–5 cm deep. A total of 152 foot samples, 38 gingival crevice samples and 88 soil samples were collected (see Supplementary Table S1).

### Study 2

**Figure 2:**
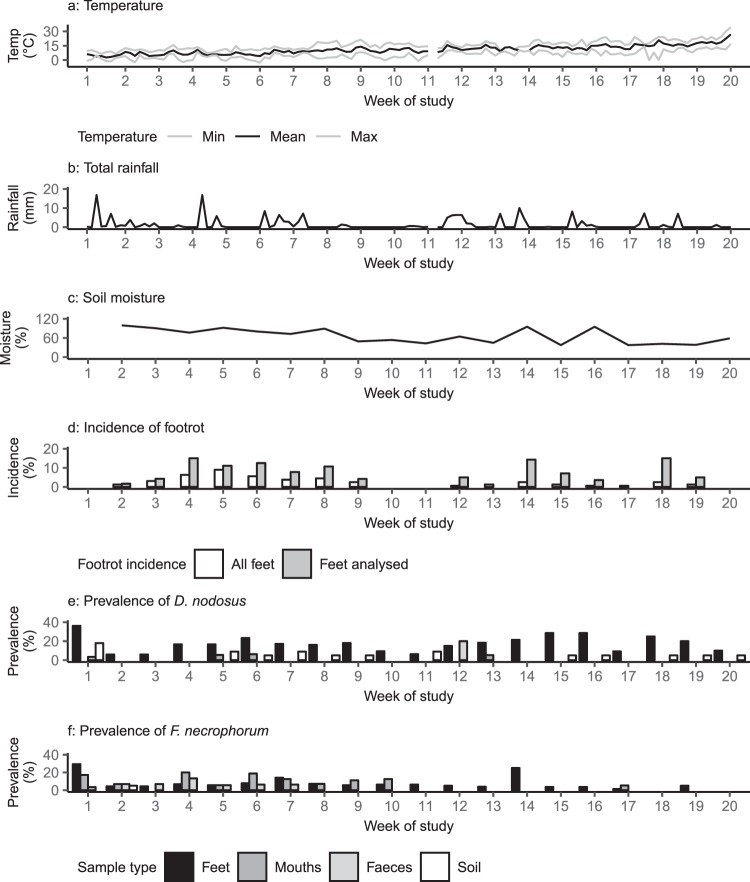
(**a**) Temperature; (**b**) total rainfall; (**c)** soil moisture; (**d**) incidence of footrot; (**e**) prevalence of *Dichelobacter nodosus*; (**f)** *Fusobacterium necrophorum* by week for Study 2. (**a**) Temperature (°C). Black = mean, grey = minimum and maximum temperatures; (**b**) total rainfall (mm). Temperature and rainfall not available for one day between weeks 11 and 12; (**c**) soil moisture (%); (**d**) incidence of footrot as a percentage of all 160 feet examined (white bars) and for feet with samples analysed (grey bars); (**e**) prevalence of *Dichelobacter nodosus* by week for Study 2 as a percentage of the number of samples analysed for each sample type**;** (**f**) prevalence of *F. necrophorum* by week for Study 2 as a percentage of the number of samples analysed for each sample type.

Study 2 was conducted on a different lowland (altitude approximately 79 meters above sea level and agricultural land classification 2 and 3^36^) commercial sheep farm in Warwickshire, England located approximately 15 miles from Study 1. The study ran during a period of prolonged dry weather with zero/low transmission of footrot (Fig. 2). The study population was a group of 120 Suffolk x Wiltshire Horn ewe lambs aged 10 months at the start of the study. All 120 ewe lambs were inspected and 40 with an ID score ≤1 and no SFR lesions^38^ were selected as the study sample: 18/40 ewe lambs had ID score 1. The 40 ewe lambs were immediately moved to the study pasture (day 1) which had not been grazed for the previous 10 days. The study was conducted for 20 weeks from February to July 2015. Each week, the 40 sheep and their pasture were sampled as for Study 1, in addition, up to 5 g of faeces was collected from the rectum of each sheep, and one extra soil sample was collected from low and high traffic sites at both depths to investigate soil moisture. All samples were collected by RC and KG. A total of 3192 foot samples, 798 gingival crevice samples, 798 faecal samples and 440 soil samples were collected (see Supplementary Table S1).

For both studies daily mean, minimum and maximum air temperature and total rainfall were sourced from the Warwick weather station (http://warwick-weather.com; last accessed August 2015).

### Laboratory analyses

#### Soil moisture estimation

Soil samples were weighed and then dried at 110 °C for 24 hours. Samples were re-weighed and soil moisture (%) was calculated using the following formula:

$$MC \% =\frac{{W}_{2}-{W}_{3}}{{W}_{3}-{W}_{1}}\times 100$$

where *MC%* = moisture content (%), *W*_1_ = weight of soil container (g), *W*_2_ = weight of moist soil + container (g), and *W*_3_ = weight of dried soil + container (g).

#### Selection of samples for analysis

A foot was defined as healthy when SFR score = 0 and ID ≤1^38^; otherwise it was defined as diseased with footrot. All samples were analysed from study 1^39,40^. From Study 2, all soil samples and a selection of sheep samples were analysed. There were 17 sheep with footrot for >1 consecutive week at least once, samples from these sheep were analysed for weeks 1–3, 5, 9, 13 and 17 and from 2 weeks before to 2 weeks after an episode of footrot or to the end of the study if this was shorter. There were 2 sheep with ID score = 0 and SFR score = 0^38^ throughout the study that were selected as control sheep and samples were analysed for weeks 1–3, 5, 9, 13 and 17. The remaining 21 sheep either had footrot once at one visit or were healthy throughout the study; 10 of these sheep were randomly selected and their samples were analysed from weeks 1–3 to establish the loads of bacteria across the cohort of 40 sheep at the start of the study. *F. necrophorum* was detected in faeces from one of these 10 sheep (Sheep ID 3520) in three consecutive weeks^1–3^, and so samples from weeks 4 and 5 were also analysed. A total of 1,100 foot swabs, 275 mouth swabs and 274 faecal samples from the 29 sheep were processed. Samples were randomised before processing by generating a random permutation of the list of samples^41^ to avoid the risk of processing bias.

#### DNA extractions from feet, mouth, faeces, and soil samples

DNA was extracted from all samples using the hydroxyapatite spin-column method^42^ with an additional polyethylene glycol precipitation step for environmental and faecal samples to further purify DNA^43^. Samples were extracted in batches of 16 with a negative control (500 μl sterile phosphate-buffered saline) in each batch.

#### Quantitative PCR for *Dichelobacter nodosus* and *Fusobacterium necrophorum*

The RNA polymerase sigma-70 factor gene (*rpoD*: present as a single copy in the *D. nodosus* genome) and the RNA polymerase subunit beta gene (*rpoB*: present as a single copy in the *F. necrophorum genome*) were targeted for the detection and enumeration of the bacterial load of *D. nodosus* and *F. necrophorum* respectively^18,44^. Details on assay sensitivities and specificities have been described previously^18,44^. The *D. nodosus* assay targets a 61 bp sequence of the *rpoD* gene using a forward primer 5′-GCTCCCATTTCGCGCATAT-3′, reverse primer 5′-CTGATGCAGAAGTCGGTAGAACA-3′ and a TaqMan® probe 5′-6FAM-TCGAACATCTCTCGCTTTTTCCCCGA-BBQ-3′. The *D. nodosus* thermal cycling conditions consisted of 1 cycle at 50 °C for 2 min, 1 cycle at 95 °C for 10 min followed by 40 cycles at 95 °C for 15 s and the final stage at 55 °C for 1 min. The *F. necrophorum* assay targets an 86 bp sequence of the *rpoB* gene using forward primer 5′-AAC CTC CGG CAG AAG AAA AAT T-3′, reverse primer 5′-CGT GAG GCA TAC GTA GAG AAC TGT-3′and TaqMan® probe 5′-6FAM-TCG AAC ATC TCT CGC TTT TTC CCCGA-BBQ-3′. The *F. necrophorum* cycling conditions consisted of an initial denaturation at 95 °C for 20 s, followed by 40 cycles of 95 °C for 30 s and a final stage at 61 °C for 30 s. All qPCR reactions were carried out in a final volume of 25 μl using 2 × TaqMan^®^ Universal Mastermix (Applied Biosystems, Thermofisher Scientific, Warrington, UK). Every qPCR plate included samples for analysis in triplicate, standard curve samples and a non-template control (sterile water). Samples were classified as positive for *D. nodosus* or *F. necrophorum* when all three qPCR replicates were positive. Thirty foot swabs from Study 2 were excluded from analysis because of contamination during processing.

#### Multiple locus variable number tandem repeat analysis (MLVA) for *Dichelobacter nodosus* and *Fusobacterium necrophorum* (Study 2 only)

In Study 2, MLVA analyses^40,45^ were performed on foot, mouth and faecal samples which were positive by qPCR. Briefly, The *D. nodosus* MLVA uses the four variable number tandem repeat (VNTR) loci DNTR02, DNTR09, DNTR10 and DNTR19 that produce a 5, 108, 48 and 84 bp sequence respectively for every tandem repeat (TR) present in the genome. The *F. necrophorum* MLVA uses the three VNTR loci Fn13, Fn42 and Fn69 that produce a 463, 465 and 458 bp sequence respectively for each TR present.

The detection limits were 10^3^ genome copies μl^−1^ template DNA for the *D. nodosus* scheme and 10^4^ genome copies μl^−1^ template DNA for the *F. necrophorum* scheme. DNA samples that produced visible amplicons on 1% agarose gel for all variable number tandem repeat (VNTR) loci were submitted for fragment analysis. The number of MLVA variants at a locus were analysed using Peak Scanner 2 software (Applied Biosystems, Thermofisher Scientific, Warrington, UK). An MLVA profile was complete when MLVA variants were identified at all loci and partial when variants were not detected at one or more loci. Each unique pattern of MLVA variants with complete MLVA profiles was assigned a unique ‘community type’ number consistent with those published previously^40,45^ and new community types were assigned a new number. Where complete MLVA profiles were present, the minimum number of strains in a sample was calculated as equal to the greatest number of MLVA variants at one locus. The maximum number of strains potentially present in a sample was calculated by multiplying the number of variants at each locus together^45^. For both complete and partial MLVA profiles, the variants present at each locus were plotted by week, sheep and foot to facilitate visual analysis of patterns over time.

### Data analysis

A new case of footrot was defined as occurrence of footrot in a foot which was healthy at the previous visit. Consequently, the incidence was the number of new cases of footrot/number of feet examined at that visit and the prevalence of footrot was the number of all cases of footrot observed/the number of all feet examined at that visit. To investigate associations with disease by foot and sheep, feet were defined as H/H = a healthy foot, all 4 feet of sheep healthy; H/D = a healthy foot, at least one other foot of the same sheep diseased; D = a diseased foot.

The frequency of *D. nodosus*/*F. necrophorum* in soil, feet, mouths and faeces was calculated from the number of qPCR positive samples/number of samples of that type for that visit. Mixed effects linear regression models^46,47^ (See Supplemental Material for details of methods for regression models) were used to investigate the mean log_10_(load +1) of *D. nodosus* and *F. necrophorum* on feet by disease state. For study 2, mixed effects binomial logistic regression models were also used to investigate the association between the presence of *D. nodosus* and *F. necrophorum* on feet and bacterial load of *D. nodosus* and *F. necrophorum* in the previous week.

Transient contamination by a bacterium was defined as a positive site on <2 and >0 consecutive visits. Persistence of a bacterium was defined as >1 consecutive visit where *D. nodosus*/*F. necrophorum*/species, strain or community was detected from the same foot, mouth or faecal sample. For Study 2, one negative sample in a period of persistence was considered a failure of detection and the sample was assumed to be positive (this occurred on 7, 1, 7 occasions for *D. nodosus, F. necrophorum* and communities, respectively).

The relationship between duration of detection of bacteria on feet and disease state was compared using mixed effects Poisson regression models^47–49^ and the predicted duration of detection of *D. nodosus*/*F. necrophorum* and associated 95% confidence intervals (CI) by disease state were calculated from the outputs of the Poisson models^50^. Significance was set at a p value ≤ 0.05 for all analyses.

## Results

### Study 1: Presence and persistence of *Dichelobacter nodosus* and *Fusobacterium necrophorum* by qPCR in a period of footrot transmission

Footrot was detected throughout Study 1. The incidence of footrot peaked at week 5 (17.5% feet; Fig. 1). Rainfall was high at the start of the study, and reduced as the study progressed. Both *D. nodosus* and *F. necrophorum* were detected on feet, throughout Study 1 (Fig. 1), and persisted on feet for 2 to at least 6 weeks. Similar to previous studies *D. nodosus* and *F. necrophorum* loads on feet were significantly higher on D feet than on H/H feet^18,33^ (Supplementary Figs S3 and S4). *D. nodosus* was detected in soil throughout the study (Fig. 1). It was detected in 41% (36/88) of soil samples with similar prevalence in high and low traffic areas (35% and 48% respectively), and surface and deep soil (36% and 45% respectively). In contrast, *F. necrophorum* was only detected in 8% (7/88) of soil samples; six at week 1 and one at week 3 and 6/7 positive samples were in surface soil from high traffic areas. *D. nodosus* was detected transiently in the mouths of 7/10 sheep whereas *F. necrophorum* was detected in mouth samples from all 10 sheep and persisted for 2 to at least 6 weeks in 9/10 sheep.

### Study 2: Presence and persistence of *Dichelobacter nodosus* and *Fusobacterium necrophorum* by qPCR and MLVA in a period of low footrot transmission

In Study 2, sheep were selected to be free from footrot (ID score ≤1)^36^ at the start of the study. Footrot was detected for the first time at week 2 and the incidence of footrot was highest in week 5 (9% feet; Fig. 2). There was no footrot (new or existing cases) at week 11, following a four-week period of very low rainfall and dry soil and incidence and prevalence of footrot remained low after this point (maximum weekly prevalence 4.4% feet, maximum weekly incidence 2.5% feet). Only 13/160 feet had a new case of footrot after week 11.

There were fewer detections of *D. nodosus* and *F. necrophorum* in Study 2 than Study 1: only 9.5% and 5.6% of all samples were positive in Study 2 compared with 53.0% and 33.6% in Study 1 respectively. The highest frequencies of detection of *D. nodosus* and *F. necrophorum* on feet were in week 1 despite all feet being healthy: 42/112 (38%) and 34/112 (30%) feet were positive respectively. Frequency of detection fell in week 2 with 7/116 (6%) and 5/116 (4%) feet positive respectively (Fig. 2).

### Presence and persistence of *Dichelobacter nodosus*

**Figure 3:**
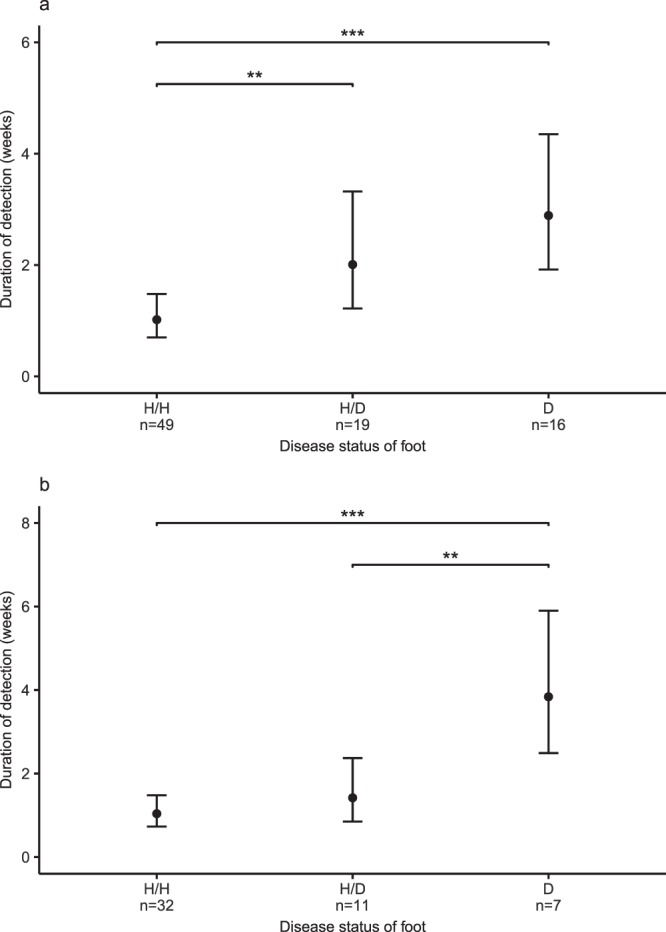
Predicted duration of detection of (**a**) *Dichelobacter nodosus* and (**b**) *Fusobacterium necrophorum* on foot swabs from 17 sheep with footrot in Study 2. Predicted duration of detection in weeks (black circles) and associated 95% confidence interval (error bars) from mixed effects Poisson regression models. H/H = healthy foot, all 4 feet of sheep healthy; H/D = healthy foot, at least one other foot of the same sheep diseased with footrot; D = diseased with footrot. ***p < 0.001; **p < 0.01. Significance levels obtained from mixed effects Poisson regression models.

In Study 2, *D. nodosus* was detected intermittently in 4.1% (18/440) of soil samples, typically when the prevalence of *D. nodosus* was highest on feet (Fig. 2). As in Study 1, there were similar percentages of *D. nodosus* positive soil samples from high and low traffic areas (3.8% and 5.0% respectively), and from surface and deep soil (5.0% and 3.6% respectively). *D. nodosus* was detected on feet in all weeks of Study 2 (Fig. 2) and persistently on 22 occasions on feet of 9 of the 17 sheep that had footrot. The vast majority (18/22) of episodes of persistence occurred on H/D and D feet (Table 1). From mixed effects Poisson regression analysis, the predicted duration of detection of *D. nodosus* was 1.02 (95% CI 0.70–1.49), 2.01 (95% CI 1.22–3.32) and 2.89 (95% CI 1.92–4.35) consecutive weeks on H/H, H/D and D feet respectively. The duration of detection was significantly longer in H/D and D feet than H/H feet (Fig. 3 and Supplementary Table S4). The H/H feet from the two control sheep were never positive for *D. nodosus* after week 1 and so played no role in its persistence (Supplementary Fig. S1). *D. nodosus* was detected transiently in the mouths of only 3/29 sheep and the faeces of 2/29 sheep.

**Table 1:** Duration of detection of *Dichelobacter nodosus* and *Fusobacterium necrophorum* on feet by disease status, 17 sheep observed for 20 weeks (Study 2).

Type of detection^*a*^	Disease status^*b*^	N^o^. episodes	N^o^. feet n = 68	N^o^. sheep n = 17	Duration^*c*^ median (range)
***D. nodosus***					
Persistent (N = 22)	H/H	4	4	3	2 (2–2)
	H/D	9	7	3	5 (3–10)
	D	9	9	7	4 (2–18)
Transient (N = 62)	H/H	45	35	17	1
	H/D	10	8	7	1
	D	7	7	6	1
No detection (NA)	Never diseased	NA	8	4	0
	Ever diseased	NA	12	9	0
***F. necrophorum***					
Persistent (N = 12)	H/H	2	2	2	2 (2–2)
	H/D	4	4	3	2 (2-3)
	D	6	6	5	3 (2–13)
Transient (N = 38)	H/H	30	26	12	1
	H/D	7	7	5	1
	D	1	1	1	1
No detection	Never diseased	NA	10	7	0
	Ever diseased	NA	21	12	0

^*a*^Persistent = Bacterium detected >1 consecutive week; Transient = Bacterium detected <2> 0 consecutive weeks; No detection = never detected during study; N = total number of episodes.

^*b*^H/H = healthy foot, all 4 feet of sheep healthy; H/D = healthy foot, at least one other foot of the same sheep diseased; D = diseased foot; Never diseased = foot never had footrot during study; Ever diseased = foot had footrot on at least one occasion during study.

^*c*^Duration = number of weeks with positive samples.

There were 88 (47%) complete and 40 (21%) partial MLVA profiles from 187 *D. nodosus* positive foot swabs. There was no amplification of VNTRs in mouth and faecal samples.

**Figure 4:**
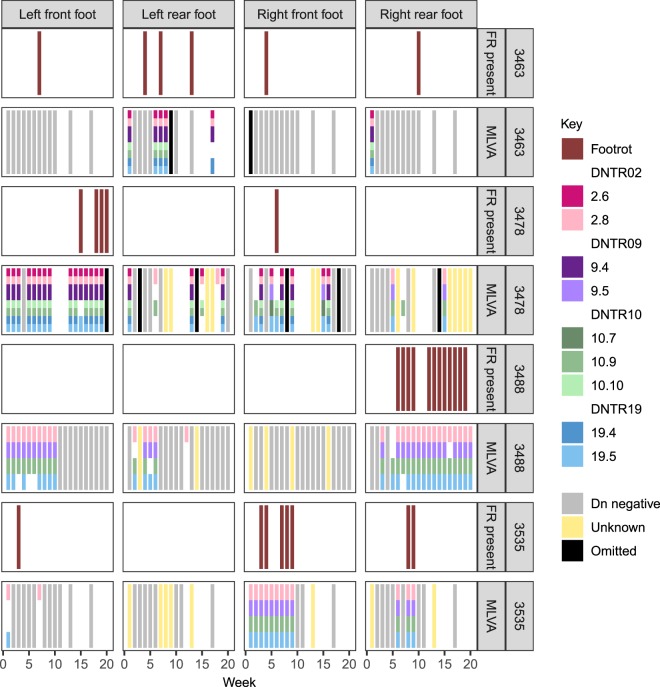
Presence of footrot and *Dichelobacter nodosus* MLVA variants by foot and week for four sheep from Study 2. Four sheep shown are those with the same MLVA community type present on ≥2 feet at the same time point as described in Results. Right of panel: 3*** = sheep identification, FR present: bar shows when footrot present, MLVA = MLVA profile. Key shows colour coding for MLVA profiles: Unknown = positive for *D. nodosus* but no MLVA variants identified. Dn negative = sample negative for *D. nodosus*.

There were eight *D. nodosus* community types detected from foot swabs (numbered 5, 19, 23–28) and between 1 and ≤8 strains of *D. nodosus* were detected per foot (Supplementary Table S8). CT5 was the only single strain community detected, and this was the most common community type accounting for 45/87 full MLVA profiles. In five sheep this was the only community type detected (Supplementary Fig. S5) and it was detected throughout the study. Of the 7 multi-strain communities, CT19 and CT23 (which included CT5) were the most frequently detected (7/87 and 27/87 full MLVA profiles respectively) whilst the other 5 multi-strain communities were only detected on 1–3 occasions. CT5 was detected on the feet of 8 sheep, CT19 was detected on feet of 2 sheep, and CT23 was detected on feet of 3 sheep. CT5, CT19 and CT23 persisted on feet for 2 to 15 weeks and accounted for 12/22 episodes of persistence of *D. nodosus* on 11 feet of 7 sheep. There were 2 feet of 2 sheep where the community type changed during an apparent episode of persistence. There were insufficient MLVA results to investigate the remaining episodes of persistence. In four sheep, the same community type was present on several feet at the same time, suggesting spatial co-location and transmission of bacteria between feet within sheep. The same community was repeatedly detected on several feet of sheep over time. (Fig. 4).

#### Presence and persistence of *Fusobacterium necrophorum*

In Study 2, *F. necrophorum* was detected in only one soil sample (n = 440), a surface sample at week 2 (Fig. 2). *F. necrophorum* was detected persistently on 12 occasions on the feet of 9 of the 17 sheep that had footrot during the study; as with *D. nodosus*, the majority (10/12) of episodes of persistence occurred on H/D and D feet (Table 1). The duration of detection was significantly longer in D feet than H/H and H/D but not between H/H and H/D feet; 1.04 (95% CI 0.73–1.48), 1.42 (95% CI 0.84–2.37) and 3.84 (95% CI 2.49–5.90) consecutive weeks on H/H, H/D and D feet respectively. (Fig. 3 and Supplementary Table S5). As with *D. nodosus*, feet of the two sheep that were always healthy were never positive for *F. necrophorum* after week 1 and so played no role in its persistence (Supplementary Fig. S2). The load of *F. necrophorum* from foot swabs was significantly higher on D feet than on H/H feet (Supplementary Fig. S4). There were 2/29 sheep where *F. necrophorum* was detected in faeces for 4 and 7 consecutive weeks. Eight of 29 sheep had at least one mouth sample positive for *F. necrophorum* and in 3 sheep it persisted for 2 to 6 weeks.

There were 24 (26%) complete and 20 (22%) partial MLVA profiles from 93 *F. necrophorum* positive foot swabs. There were 4 (19%) complete and 13 (62%) partial MLVA profiles from 21 positive mouth swabs and 2 complete and 8 partial MLVA profiles from 10 positive faecal samples. Five *F. necrophorum* community types were detected (types 1, 8, 9, 18, and 19; Supplementary Table S9). One strain of *F. necrophorum* was detected from all samples with complete MLVA profiles with the exception of one foot swab where a community of 3 strains was detected (CT19). The same single strain community (CT8) was detected on 22/24 foot swabs and this community type persisted for 2 weeks on 3 feet of 2 sheep, and for 10 weeks on one foot of one sheep (Supplementary Fig. S6). The MLVA partial profiles indicated that strains of *F. necrophorum* were persistently shed in faeces for at least 4 weeks (Supplementary Fig. S7). There was evidence of strain specificity of *F. necrophorum* by site; strains in mouths and on feet were also detected in faeces, however, strains on feet were never detected in mouths and vice versa (Supplementary Figs S6 and S7).

#### Association between presence of *Dichelobacter nodosus* and *Fusobacterium necrophorum*

Feet were more likely to be positive for *F. necrophorum* when the load of *D. nodosus* was higher the previous week (OR 1.52, 95% CI 1.25–1.89), however there was no significant association between the presence of *D. nodosus* and the load of *F. necrophorum* the previous week (see Supplementary Tables S6 and S7 for full model results), indicating that *D. nodosus* initiated colonisation of *F. necrophorum*.

## Discussion

This is the first study to use molecular tools to investigate *D. nodosus* and *F. necrophorum* directly in the host and environment. Our findings represent a step change in understanding of the sites of persistence of these pathogens that inform on the epidemiology of footrot and enable us to evaluate the potential for elimination of *D. nodosus* and *F. necrophorum* from sheep flocks.

A key paradigm shift is that *F. necrophorum*, far from being ubiquitous in soil, is rarely present in soil (Figs 1 and 2) and is present and shed in faeces of only a small number of sheep (2/29 in Study 2; Fig. 2). Where *F. necrophorum* was detected in soil, it was in wet conditions, high traffic areas and at the surface (Figs 1 and 2), all indicative of very transient contamination. We therefore conclude that *F. necrophorum* is, in fact, highly host dependent, shed into the environment by a few animals and does not persist off host. Our results are in contrast to perceived beliefs of ubiquity, but do provide an explanation for some published reports of *F. necrophorum*-related disease. For example, outbreaks of interdigital necrobacillosis, caused by *F. necrophorum*, occur in cattle and reindeer when animals are crowded together during periods of high rainfall^51,52^ and crowding of sheep in faecally contaminated conditions results in colonisation of the interdigital skin with *F. necrophorum*^53^. Those reports are compatible with a few animals shedding *F. necrophorum* in faeces which, in wet conditions, provide a medium for bacteria to survive, circulate and transmit to many animals in the group. At the first visit of Study 1 it was raining and so where the sheep were gathered the conditions for *F. necrophorum* transmission were present, this is reflected by the high prevalence of *F. necrophorum* detected on feet at visit 1 (Fig. 1).

The detection of *D. nodosus*, but not *F. necrophorum*, in low traffic areas and deep soil samples suggests that *D. nodosus* survives longer in the environment off host than *F. necrophorum*. *D. nodosus* was detected in soil in all weeks in Study 1 and 12/20 weeks in Study 2. The results highlight the role of moisture in the survival of *D. nodosus* off host, the wet weather in Study 1 facilitated persistence of *D. nodosus* in the environment, and enabled transmission of *D. nodosus* between feet and sheep. *D. nodosus* was not detected on pasture for several weeks after the onset of dry weather in the middle of Study 2 (Fig. 2) indicating that *D. nodosus* did not persist on dry soil and that it does not persist for many days on pasture. *D. nodosus* has only a small genome and cannot synthesise all 20 amino acids^54^ so it is likely that survival of *D. nodosus* in soil is limited to a few days. We conclude from the results in our studies that soil is repeatedly contaminated with *D. nodosus* shed from infected/diseased feet. Therefore, pasture can be a constant source of infection when *D. nodosus-*positive feet are present or for a short period after sheep have been removed but *D. nodosus* does not persist on dry pasture or in the absence of sheep. This is compatible with a report by Whittington^55^ that footrot occurs in naïve sheep placed in contaminated environments following the absence of sheep for a short period. Given the duration of persistence of *D. nodosus* on pasture in the two studies presented here (Figs 1 and 2), we conclude that persistent contamination of pasture is common in the UK climate where damp pasture is highly prevalent.

We have demonstrated that strains of *D. nodosus* persist on footrot affected feet for prolonged periods of time and that the same strains of *D. nodosus* persisted on some healthy feet of sheep with footrot (H/D), however, *D. nodosus* did not persist on healthy feet of healthy sheep (Table 1 and Figs 3 and 4). We conclude that this is likely to be repeated cross contamination by diseased feet because of spatial co-location rather than prolonged persistence, because healthy feet of healthy sheep do not have prolonged persistence of *D. nodosus*. Therefore, we conclude that *D. nodosus* only persists on feet diseased with footrot, including mild interdigital dermatitis and inactive footrot that might not cause lameness^3,56^ which highlights that in periods of non-transmission diseased feet could remain unnoticed within a flock unless all feet are inspected^12,57,58^. Given our evidence that *D. nodosus* cannot persist in soil in the absence of footrot, and the lack of evidence for carriage in faeces, mouth or healthy feet (Supplementary Table S3), we conclude that removal of all sheep with footrot from a flock during a period of zero transmission should result in elimination of *D. nodosus*. This was postulated by Beveridge^8^, and demonstrated in individual flock level elimination programmes in Australia, UK and Nepal^10,59–62^ which used periods of zero transmission together with control measures to reduce the load of *D. nodosus* including vaccination, treatment, culling and footbathing.

Our analysis indicated that *F. necrophorum* colonised feet after *D. nodosus* and that load and persistence were only significantly greater in footrot affected feet (Supplementary Figs S3 and S4, Table 1). This adds weight to the evidence that *F. necrophorum* is acting opportunistically as a secondary coloniser. *F. necrophorum’s* site of persistence in the absence of footrot is in faeces of a few sheep. Faecal persistence could occur through a few sheep that persistently carry and shed *F. necrophorum* or through all sheep shedding *F. necrophorum* for short periods of time rather than persistently, the latter is the suggested route for persistence for *E. coli* in cattle^63^. Whichever is true, the presence of *F. necrophorum* in faeces provides a mechanism by which *F. necrophorum* persists within a sheep flock, and can be transferred between flocks, in the absence of footrot. This would make elimination of *F. necrophorum* from sheep flocks very difficult. If the former is true it would be possible to test and remove persistent carriers, however, if the latter is true and all sheep, rather than certain sheep, shed *F. necrophorum* intermittently in faeces then elimination of *F. necrophorum* would not be possible. Strain communities of *F. necrophorum* detected in mouths were different from those on feet and so may not be linked to footrot. If *F. necrophorum* strains exhibit site specificity as suggested here (Supplementary Figs S6 and S7) and in Clifton *et al*.^45^, where only a few strains were detected on feet,  it might be possible to eliminate only the foot-related strains of *F. necrophorum*.

Across the two studies a total of 5,698 samples were collected and 2,227 samples were analysed from two flocks on lowland farms with similar altitude and agricultural land classification but with different patterns of rainfall and temperature. Different sites of persistence were identified for *D. nodosus* and *F. necrophorum* and these sites of persistence were detected in both flocks despite the differences in rainfall and temperature. These consistent findings between studies indicate that the results are robust and that our conclusions are likely to be generalisable to flocks in temperate climates. Our results are entirely compatible with other studies that have reported the elimination of *D. nodosus* in arid climates^61,62^ i.e. *D. nodosus* does not persist on pasture and so there is no transmission of bacteria. There is insufficient published work on *F. necrophorum* to postulate how this organism’s behaviour would change in arid conditions and whether it could be eliminated but if the sites of persistence are the same, our results would suggest the possibility of elimination is unlikely. Our results highlight the value of MLVA community typing to investigate persistence in non-cultured DNA samples. In Study 2 we did not analyse samples from all 40 sheep, however, within the sheep we selected we had representation of feet and sheep in all disease states (H/H, H/D and D). Given that *D. nodosus* and *F. necrophorum* were primarily detected on the feet of sheep with footrot, and the 11 sheep we did not analyse had very little or no footrot, and so would probably be negative for the pathogens, it is unlikely that analysis of these samples would have changed our results.

In conclusion, through direct study of host, pathogen and environment we determined that *D. nodosus* and *F. necrophorum* have distinct patterns of persistence in sheep flocks. The fastidious and highly specialised pathogen, *D. nodosus*, which persists for long periods on diseased feet, and spreads by persisting for short periods on healthy feet and pasture depends on disease expression to persist in flocks and so elimination of *D. nodosus* through elimination of all severities of footrot, as described by Beveridge^8^ is possible. In contrast to previous reports, the presumed ubiquitous, opportunistic pathogen, *F. necrophorum*, is also highly host specific, persisting in faeces and footrot affected feet and rarely detected on pasture. *F. necrophorum* is not dependent on footrot expression to persist in flocks because it persists in faeces and so elimination is highly unlikely to be possible. Given that *F. necrophorum* is a secondary pathogen, control of footrot should focus on the elimination of *D. nodosus* from sheep through total removal of clinical signs of footrot and *D. nodosus* from the environment.

## Supplementary information

Supplementary material

## Data Availability

The datasets generated during and/or analysed during the current study are available from the corresponding author on reasonable request. Codes generated are also available on request.
