# Supplementary material for: Sites of persistence of Fusobacterium necrophorum and Dichelobacter nodosus: a paradigm shift in understanding the epidemiology of footrot in sheep
Source: Sci Rep. 2019 Oct 8;9:14429. doi: 10.1038/s41598-019-50822-9 (PMC6783547; doi:10.1038/s41598-019-50822-9)
Supplement: Supplementary file 1 — Supplementary material [file 41598_2019_50822_MOESM1_ESM.docx]

**Sites of persistence of *Fusobacterium necrophorum* and *Dichelobacter nodosus*: a paradigm shift in understanding the epidemiology of footrot in sheep. Supplementary material**

Rachel Clifton, Katharina Giebel, Nicola L.B.H. Liu, Kevin J. Purdy and Laura E. Green

**Table S1.** Number of samples collected by site by visit and in total for two studies

| Study | N^o^. of visits | Feet  (total) | Mouth  (total) | Faeces  (total) | Soil  (total) |
| --- | --- | --- | --- | --- | --- |
| 1 | 4 | 32-40* (152) | 8-10 (38) * | NA | 22 (88) |
| 2 | 20 | 160** (3192) | 40 (798) ** | 40 (798) ** | 22 (440) |

* Two lambs were not present on the farm at visit 4. ** On two occasions a sheep escaped and was not sampled. NA = not applicable.

**Table S2.** Detection frequency of *D. nodosus* and *F. necrophorum* by week and sample type in Study 1

| Week | Foot swabs  (n=40*) | | Mouth swabs (n=10*) | | Soil  (n=22) | |
| --- | --- | --- | --- | --- | --- | --- |
|  | N^o^. | % | N^o^. | % | N^o^. | % |
| *D. nodosus* | |  |  |  |  |  |
| 1 | 40 | 100 | 4 | 40 | 19 | 86 |
| 3 | 27 | 68 | 1 | 10 | 9 | 41 |
| 5 | 18 | 45 | 3 | 30 | 5 | 23 |
| 7 | 16 | 50 | 2 | 25 | 3 | 14 |
| Overall | 101 | 66 | 13 | 33 | 36 | 42 |
| *F. necrophorum* | |  |  |  |  |  |
| 1 | 39 | 98 | 9 | 90 | 6 | 27 |
| 3 | 17 | 43 | 9 | 90 | 1 | 5 |
| 5 | 11 | 28 | 6 | 60 | 0 | 0 |
| 7 | 9 | 28 | 6 | 75 | 0 | 0 |
| Overall | 76 | 50 | 30 | 79 | 7 | 8 |

*Only 8 sheep were sampled in week 7 therefore a total of 32 foot swabs and 8 mouth swabs were taken. No. = number of positive samples, % = percentage of positive samples.

**Table S3.** Detection frequency of *D. nodosus* and *F. necrophorum* by week and sample type in Study 2

| Week | Foot swabs | | Mouth swabs | | Soil | | Faeces | |
| --- | --- | --- | --- | --- | --- | --- | --- | --- |
|  | N^o^ | % | N^o^. | % | N^o^. | % | N^o^. | % |
| *D. nodosus* |  |  |  |  |  |  |  |  |
| 1 | 42/112 | 38 | 0/29 | 0 | 4/22 | 18 | 1/29 | 3 |
| 2 | 7/116 | 6 | 0/29 | 0 | 0/22 | 0 | 0/29 | 0 |
| 3 | 7/114 | 6 | 0/29 | 0 | 0/22 | 0 | 0/29 | 0 |
| 4 | 10/59 | 17 | 0/15 | 0 | 0/22 | 0 | 0/15 | 0 |
| 5 | 12/72 | 17 | 1/18 | 6 | 2/22 | 9 | 0/18 | 0 |
| 6 | 15/64 | 23 | 1/16 | 6 | 1/22 | 5 | 0/16 | 0 |
| 7 | 11/61 | 18 | 0/16 | 0 | 2/22 | 9 | 0/16 | 0 |
| 8 | 9/52 | 17 | 0/14 | 0 | 1/22 | 5 | 0/14 | 0 |
| 9 | 13/68 | 19 | 0/18 | 0 | 1/22 | 5 | 0/18 | 0 |
| 10 | 3/31 | 10 | 0/8 | 0 | 0/22 | 0 | 0/8 | 0 |
| 11 | 1/16 | 6 | 0/4 | 0 | 2/22 | 9 | 0/4 | 0 |
| 12 | 3/20 | 15 | 0/5 | 0 | 0/22 | 0 | 1/5 | 20 |
| 13 | 14/75 | 19 | 1/19 | 5 | 0/22 | 0 | 0/18 | 0 |
| 14 | 6/25 | 24 | 0/7 | 0 | 0/22 | 0 | 0/7 | 0 |
| 15 | 8/28 | 29 | 0/7 | 0 | 1/22 | 5 | 0/7 | 0 |
| 16 | 8/26 | 31 | 0/7 | 0 | 1/22 | 5 | 0/7 | 0 |
| 17 | 7/73 | 10 | 0/19 | 0 | 0/22 | 0 | 0/19 | 0 |
| 18 | 5/19 | 26 | 0/5 | 0 | 1/22 | 5 | 0/5 | 0 |
| 19 | 4/20 | 20 | 0/5 | 0 | 1/22 | 5 | 0/5 | 0 |
| 20 | 2/19 | 11 | 0/5 | 0 | 1/22 | 5 | 0/5 | 0 |
| Overall | 187/1070 | 17 | 3/275 | 1 | 18/440 | 4 | 2/274 | 1 |
| *F. necrophorum* | |  |  |  |  |  |  |  |
| 1 | 34/112 | 30 | 5/29 | 17 | 0/22 | 0 | 1/29 | 3 |
| 2 | 5/116 | 4 | 2/29 | 7 | 1/22 | 5 | 2/29 | 7 |
| 3 | 5/114 | 4 | 0/29 | 0 | 0/22 | 0 | 2/29 | 7 |
| 4 | 4/59 | 7 | 3/15 | 20 | 0/22 | 0 | 2/15 | 13 |
| 5 | 4/72 | 6 | 1/18 | 6 | 0/22 | 0 | 1/18 | 6 |
| 6 | 5/64 | 8 | 3/16 | 19 | 0/22 | 0 | 1/16 | 6 |
| 7 | 9/61 | 15 | 2/16 | 13 | 0/22 | 0 | 1/16 | 6 |
| 8 | 4/52 | 8 | 1/14 | 7 | 0/22 | 0 | 0/14 | 0 |
| 9 | 4/68 | 6 | 2/18 | 11 | 0/22 | 0 | 0/18 | 0 |
| 10 | 2/31 | 6 | 1/8 | 13 | 0/22 | 0 | 0/8 | 0 |
| 11 | 1/16 | 6 | 0/4 | 0 | 0/22 | 0 | 0/4 | 0 |
| 12 | 1/20 | 5 | 0/5 | 0 | 0/22 | 0 | 0/5 | 0 |
| 13 | 3/75 | 4 | 0/19 | 0 | 0/22 | 0 | 0/18 | 0 |
| 14 | 7/25 | 28 | 0/7 | 0 | 0/22 | 0 | 0/7 | 0 |
| 15 | 1/28 | 4 | 0/7 | 0 | 0/22 | 0 | 0/7 | 0 |
| 16 | 1/26 | 4 | 0/7 | 0 | 0/22 | 0 | 0/7 | 0 |
| 17 | 1/73 | 1 | 1/19 | 5 | 0/22 | 0 | 0/19 | 0 |
| 18 | 0/19 | 0 | 0/5 | 0 | 0/22 | 0 | 0/5 | 0 |
| 19 | 1/20 | 5 | 0/5 | 0 | 0/22 | 0 | 0/5 | 0 |
| 20 | 0/19 | 0 | 0/5 | 0 | 0/22 | 0 | 0/5 | 0 |
| Overall | 92/1070 | 9 | 21/275 | 8 | 1/440 | 0 | 10/274 | 4 |

N^o^. = number of positive samples/total samples, % = percentage of positive samples.

**Figure S1.** Study 2. Detection of *Dichelobacter nodosus* by health status of foot by week for two healthy sheep and 17 sheep with footrot

Right hand box = sheep identification. Dn = D*. nodosus*, RR = right rear, RF = right front, LR = left rear, LF = left front. Omitted = due to suspected contamination. Gaps = samples not analysed all feet were healthy.

**Figure S2.** Study 2. Detection of *Fusobacterium necrophorum* by health status of foot by week for two healthy sheep and 17 sheep with footrot

Right hand box = sheep identification. Fn = *F. necrophorum*, RR = right rear, RF = right front, LR = left rear, LF = left front. Omitted = due to suspected contamination. Gaps = samples not analysed all feet were healthy.

**Associations between footrot status and load of *F. necrophorum* and *D. nodosus* on foot swabs**

Three-level continuous outcome models were used to investigate associations between *D. nodosus* and *F. necrophorum* load on foot swabs and footrot status of feet. Feet were coded H/H = healthy foot, all 4 feet of sheep healthy; H/D = healthy foot, at least one other foot of the same sheep diseased; D = diseased foot. Separate models were constructed for Study 1 and Study 2, and for *D. nodosus* and *F. necrophorum*. Only samples positive for *D. nodosus* or *F. necrophorum* were included. Observations were grouped by foot and by sheep to take account of clustering of observations of the same foot over time, and of feet within a sheep. All models included time as an explanatory variable. For Study 1 time was coded as a categorical explanatory variable i.e. each visit was a category. For Study 2 because there were a larger number of visits, a 4^th^ order polynomial term for week of the study was used and week of the study was mean centred. The models took the form:

$$y_{ijk}=\beta_{0}+{\beta x}_{ijk}+v_{k}+u_{jk}+e_{ijk}$$

where y was the continuous outcome variable, log_10_(load + 1) of *D. nodosus* or *F. necrophorum*, *β_0_* was the intercept, and ${\beta x}_{ijk}$was the explanatory variable footrot status of feet categorised as H/H, H/D or D/D. Residual variance estimates were included at sheep (*v_k_*), foot (*u_jk_*) and observation (*e_ijk_*).

Model results are illustrated in Figures S3 and S4.

Study 1

Study 2

**Figure S3.** Boxplots of load of *D. nodosus* in foot samples by footrot status of foot

*** p<0.001; **p<0.01; *p<0.05

Study 1

Study 2

**Figure S4.** Boxplots of load of *F. necrophorum* in foot samples by footrot status of foot

*** p<0.001; **p<0.01; *p<0.05

**Associations between footrot status and duration of detection of *D. nodosus / F. necrophorum* on feet**

Mixed effects Poisson models were used to investigate associations between duration of detection of *D. nodosus* and *F. necrophorum* on feet and footrot status (classified as H/H, H/D or D) for Study 2. Separate models were constructed for *D. nodosus* and *F. necrophorum.* Initially single level Poisson models were run and checked for over-dispersion using the method by A. C. Cameron and P. K. Trivedi (1). Mixed effects Poisson models were then tested. Observations were grouped by foot and by sheep to take account of clustering of observations of the same foot over time, and of feet within a sheep. The models took the form:

$$Log\left( \pi_{ijk} \right)=\beta_{0}+{\beta x}_{ij}+v_{k}+u_{j}+e_{ij}$$

where Log($\pi_{ijk}$) was the log of the number of weeks a foot was positive for *D. nodosus* or *F. necrophorum*, *β_0_* was the intercept, and ${\beta x}_{ij}$was the explanatory variable footrot status of sheep categorised as healthy or diseased. Residual variance estimates were included at sheep (*v_k_*), foot (*u_jk_*) and observation (*e_ijk_*).

**Table S4.** Model results of mixed effects Poisson regression model to compare duration of detection of *D. nodosus* in weeks on feet in different disease states in Study 2

| Disease status of foot | Episodes of detection of *D. nodosus* (n=84) | | RR | 95% CI | |
| --- | --- | --- | --- | --- | --- |
|  | N | % |  |  |  |
| H/H | 49 | 58 | 1.00 |  |  |
| H/D | **19** | **23** | **1.96** | **1.19** | **3.31** |
| D | **16** | **19** | **2.82** | **1.80** | **4.38** |
|  |  |  |  |  |  |
| Random part |  |  |  |  |  |
| Variance (foot level) | 0.16 |  |  |  |  |
| Variance (sheep level) | 0.17 |  |  |  |  |

**Table S5** Model results of mixed effects Poisson regression model to compare duration of detection of *F. necrophorum* in weeks on feet in different disease states in Study 2

| Disease status of foot | Episodes of detection of *F. necrophorum* (n=50) | | RR | 95% CI | |
| --- | --- | --- | --- | --- | --- |
|  | N | % |  |  |  |
| D | 7 | 14 | 1.00 |  |  |
| H/H | **32** | **64** | **0.27** | **0.16** | **0.47** |
| H/D | **11** | **22** | **0.37** | **0.19** | **0.73** |
|  |  |  |  |  |  |
| Random part |  |  |  |  |  |
| Variance (foot level) | 6.20 x 10^-2^ |  |  |  |  |
| Variance (sheep level) | 4.15 x 10^-10^ |  |  |  |  |

For tables S4 and S5:

BOLD = Significant associations with duration of detection (Wald’s test p<0.05). N = number of episodes; RR = risk ratio; CI = confidence intervals

H/H = healthy foot, all 4 feet of sheep healthy; H/D = healthy foot, at least one other foot of the same sheep diseased; D = diseased foot

**Associations between the presence of *D. nodosus* / *F. necrophorum* and health status and bacterial load of *D. nodosus* and *F. necrophorum* in the previous week**

Mixed effects binomial logistic regression models were used to investigate the associations between the presence of *D. nodosus* / *F. necrophorum* and health status and bacterial load of *D. nodosus* and *F. necrophorum* in the previous week. Week of the study was mean-centred and included in the models as a fourth-degree polynomial term (week + week^2^ + week^3^ + week^4^) to account for any underlying patterns over time. The models took the form:

$$Logit\left( \pi_{ijk} \right)=\beta_{0}+{\beta x}_{ijk}{+v}_{k}+u_{jk}+e_{ijk}$$

where Logit($\pi_{ijk}$) is the log odds of the probability that a foot swab was positive for *D. nodosus* or *F. necrophorum*, *β_0_* is the intercept, and ${\beta x}_{ijk}$ are the lagged explanatory variables. The residual variance estimates at sheep (*v_k_*), and foot (*u_jk_*) are assumed to follow a normal distribution with a mean of 0 and variance of 1, and observation ($e_{ijk}$) which is restricted to a binomial distribution with variance $\frac{\pi^{2}}{3}=3.29$.

**Table S6.** Study 2: Multivariable binomial mixed effects regression model of presence of *D. nodosus* on foot swabs as determined by qPCR (n=795)

| Value one-week previously | No. | % | OR | Lower  95% CI | Upper  95% CI |
| --- | --- | --- | --- | --- | --- |
| Log_10_(Fn load + 1) | 795 | 100 | 1.13 | 0.92 | 1.39 |
| Log_10_(Dn load + 1) | **795** | **100** | **1.54** | **1.30** | **1.83** |
| Disease status D | 74 | 9 | Ref |  |  |
| Disease status H/H | 541 | 68 | 0.53 | 0.24 | 1.25 |
| Disease status H/D | **180** | **23** | **0.37** | **0.15** | **0.93** |
|  |  |  |  |  |  |
| Week* | 795 | 100 | 3.61 x 10^2^ | 5.91 x 10^-2^ | 2.05 x 10^6^ |
| Week^2^ | **795** | **100** | **1.10 x 10^-5^** | **2.47 x 10^-9^** | **3.15 x 10^-2^** |
| Week^3^ | 795 | 100 | 1.20 x 10^1^ | 3.69 x 10^-3^ | 3.66 x 10^4^ |
| Week^4^ | **795** | **100** | **9.52 x 10^-6^** | **4.64 x 10^-9^** | **1.37 x 10^-2^** |
| Random part |  |  |  |  |  |
| Variance (foot level) | <0.0001 |  |  |  |  |
| Variance (sheep level) | 0.87 |  |  |  |  |

**Table S7.** Study 2: Multivariable binomial mixed effects regression model of presence of *F. necrophorum* on foot swabs as determined by qPCR (n=795)

| Value one-week previously | No. | % | OR | Lower  95% CI | Upper  95% CI |
| --- | --- | --- | --- | --- | --- |
| Log_10_(Fn load + 1) | **795** | **100** | **1.44** | **1.15** | **1.80** |
| Log_10_(Dn load + 1) | **795** | **100** | **1.52** | **1.25** | **1.89** |
| Disease status D | 74 | 9 | Ref |  |  |
| Disease status H/H | 541 | 68 | 1.04 | 0.36 | 0.34 |
| Disease status H/D | 180 | 23 | 1.02 | 0.36 | 3.70 |
|  |  |  |  |  |  |
| Week* | 795 | 100 | 1.45 x 10^-6^ | 1.65 x 10^-17^ | 3.33 x 10^1^ |
| Week^2^ | **795** | **100** | **2.22 x 10^-11^** | **5.17 x 10^-22^** | **2.24 x 10^-4^** |
| Week^3^ | 795 | 100 | 2.65 x 10^-5^ | 7.41 x 10^-15^ | 1.74 x 10^2^ |
| Week^4^ | 795 | 100 | 6.16 x 10^-6^ | 1.25 x 10^-12^ | 1.71 x 10 |
| Random part |  |  |  |  |  |
| Variance (foot level) | 0.51 |  |  |  |  |
| Variance (sheep level) | 0.15 |  |  |  |  |

For both table S4 & S5:

* week is mean-centred for all polynomial terms

No. = number of values in the dataset, % = percentage of values in the dataset

OR = odds ratio, CI = confidence interval

Fn = *F. necrophorum*, Dn = *D. nodosus*

H/H = healthy foot, all 4 feet of sheep healthy; H/D = healthy foot, at least one other foot of the same sheep diseased; D = diseased foot

**Table S8.** Details of the composition of variants and potential number of strains for each unique MLVA community type for *D. nodosus*

| Community type | VNTR loci and the variants present | | | | | | | | | | | | Potential number of strains | |
| --- | --- | --- | --- | --- | --- | --- | --- | --- | --- | --- | --- | --- | --- | --- |
|  | DNTR02 | | | | DNTR09 | | DNTR10 | | | DNTR19 | | |  |  |
|  | 2.5 | 2.6 | 2.7 | 2.8 | 9.4 | 9.5 | 10.7 | 10.9 | 10.10 | 19.3 | 19.4 | 19.5 | Min. | Max. |
| 5 |  |  |  | ✓ |  | ✓ |  | ✓ |  |  |  | ✓ | 1 | 1 |
| 19 |  |  | ✓ | ✓ |  | ✓ |  | ✓ |  | ✓ |  | ✓ | 2 | 4 |
| 23 |  | ✓ |  | ✓ | ✓ |  |  | ✓ | ✓ |  | ✓ | ✓ | 2 | 8 |
| 24 |  | ✓ |  | ✓ |  | ✓ | ✓ |  |  |  |  | ✓ | 2 | 2 |
| 25 |  | ✓ |  | ✓ |  | ✓ | ✓ |  |  | ✓ |  |  | 2 | 2 |
| 26 |  |  |  | ✓ | ✓ |  | ✓ | ✓ |  |  |  | ✓ | 2 | 2 |
| 27 | ✓ |  |  |  | ✓ | ✓ |  | ✓ | ✓ | ✓ |  | ✓ | 2 | 8 |
| 28 |  |  |  | ✓ |  | ✓ | ✓ | ✓ |  |  |  | ✓ | 2 | 2 |

**Table S9.** Details of the composition of variants and potential number of strains for each unique MLVA community type for *F. necrophorum*

| Community type | Sample type | VNTR loci and the variants present | | | | | | | | | Potential number of strains | | |
| --- | --- | --- | --- | --- | --- | --- | --- | --- | --- | --- | --- | --- | --- |
|  |  | Fn13 | | Fn42 | | | | Fn69 | | |  | |  |
|  |  | 13.1a | 13.2 | 42.4 | 42.5 | 42.6 | 42.7 | 69.2 | 69.3 | 69.4 | Min. | Max. | |
| 1 | M, Fa |  | ✓ |  | ✓ |  |  | ✓ |  |  | 1 | 1 | |
| 8 | F, Fa |  | ✓ |  | ✓ |  |  |  | ✓ |  | 1 | 1 | |
| 9 | M |  | ✓ |  |  |  | ✓ |  | ✓ |  | 1 | 1 | |
| 18 | F | ✓ |  |  | ✓ |  |  |  |  | ✓ | 1 | 1 | |
| 19 | F |  | ✓ | ✓ | ✓ |  | ✓ |  | ✓ |  | 3 | 3 | |

F = foot swab, M = mouth swab and Fa = faecal sample.

**Figure S5.** *Dichelobacter nodosus* MLVA variants by foot and week for sheep from Study 2.

Each horizontal panel represents one sheep. Only sheep with MLVA profiles are shown. Right of panel: PCT = persistent community type, PDn = persistence of *D. nodosus* but not community type, T = transient community type/*D. nodosus*, C = control sheep. Key shows colour coding for MLVA profiles: Unknown = positive for *D. nodosus* but no MLVA variants identified. Dn negative = sample negative for *D. nodosus*.

**Figure S6.** *Fusobacterium necrophorum* MLVA variants by foot and week for sheep from Study 2.

Each horizontal panel represents one sheep. Only sheep with MLVA profiles are shown. Right of panel: PCT = persistent community type, PFn = persistence of *F. necrophorum* but not community type, T = transient community type/*F. necrophorum*. Key shows colour coding for MLVA profiles: Unknown = positive for *F. necrophorum* but no MLVA variants identified. Fn negative = sample negative for *F. necrophorum*.

**Figure S7.** Study 2. *Fusobacterium necrophorum* MLVA variants for mouth and faecal samples for sheep 3463, 3520 and 3539 by week.

Right of panel: 3*** = sheep identification. Key shows colour coding for MLVA profiles: Unknown = positive for *F. necrophorum* but no MLVA variants identified. Fn negative = sample negative for *F. necrophorum*.

**References**

1. Cameron AC, Trivedi PK. 1990. Regression-based tests for overdispersion in the Poisson model. Journal of Econometrics 46:347-364.
